# Could the severity of COVID-19 be increased by low gastric acidity?

**DOI:** 10.1186/s13054-020-03182-0

**Published:** 2020-07-22

**Authors:** Elizabeth Price

**Affiliations:** London, UK

Could low gastric acidity increase the risk of a severe COVID-19 illness? Although it is primarily a respiratory infection, gastrointestinal involvement from swallowed coronaviruses is reported for SARS-CoV-2 (the virus of COVID-19 [1, 2]), as well as SARS-CoV-1 [3] and MERS-CoV viruses [4].

The gastrointestinal tract may be a secondary route for spread to the lungs and other parts of the body. A possible hypothesis might be that the upper respiratory tract is attacked by viruses which are breathed in and coughed up in sputum while the lower respiratory tract is similarly infected, but is also attacked at the same time by blood-borne viruses (following translocation from a significant viral load in the gastrointestinal tract). The former might result in mild or moderate illnesses only. The latter may cause a more severe illness, as the lungs are being attacked by viruses coming from two routes simultaneously.

Although it can be transiently higher, the pH of normal gastric acid is generally between 1.5 and 3.5. The SARS-CoV-1 virus is inactivated at a pH < 3.0 and > 12.0 [5]. Assuming these inactivation levels are similar for SARS-CoV-2, gastric acid will not inhibit all the viruses in the stomach (and some viruses will be hidden in food boluses). However, the inhibition that does occur may be enough to decrease the viral load entering the small intestine. In many older adults, the gastric acid pH is higher than normal, either because of atrophic gastritis or because of antacid and acid-reducing medications. One oral dose of a proton pump inhibitor raises the gastric acid pH from 2.0 to over 6.0, which will not inhibit the virus [6]. (For MERS-CoV, treatment with a proton pump inhibitor in an animal model resulted in exaggerated infection in the small intestine [4].)

In a study of 204 COVID-19 patients, fever or respiratory symptoms were generally present but 50% also reported digestive symptoms, particularly appetite loss and diarrhoea [1]. In a literature search, abdominal pain was associated with a near fourfold increased odds of severe disease, possibly because of a high viral load, viral replication in the gut and viremia [2].

Children rarely suffer from severe illnesses with COVID-19. As well as protection related to immunological factors and possible differences in the ACE2 receptor concentrations in their lungs, children (other than infants) generally have good levels of gastric acid. This may provide some protection from swallowed viruses, even though young children usually swallow their sputum rather than spit it out.

To determine whether gastric acid gives some protection from COVID-19, the amount of antacids and acid-reducing drugs used by patients with severe infections could be compared with the amount used by patients with mild or no disease. It should not be overlooked that many of these medications may be purchased over-the-counter and be taken only occasionally for gastrointestinal symptoms, rather than being prescribed by a medical practitioner. Therefore, they may not appear on a list of a patient’s usual medications. Whether there is any relation between taking these drugs and the clinical course could be considered. Drugs taken during hospital admissions should also be recorded, as hospitals may continue medications taken at home. In addition, many intubated patients are given acid-reducing drugs and gastrointestinal feeding may be continuous rather than intermittent. Such factors could result in a gastric pH of around 4.0 or 5.0. This would not inactivate these viruses, which might then pass into the small intestine where the relevant ACE2 receptors are abundant.

If there is evidence for some protection by gastric acidity, stopping antacids and acid-reducing medications could be considered, particularly at times when patients are at increased risk.

## Data Availability

Not applicable

## References

[1] Yang L, Tu L. Implications of gastrointestinal manifestations of COVID-19. Lancet Gastroenterol Hepatol 2020 Published Online May 12, 2020 10.1016/S2468-1253(20)30132-1. 10.1016/S2468-1253(20)30132-1 PMC7217632 32405602

[2] Henry BM, de Oliveira MHS, Benoit J, Lippi G. Gastrointestinal symptoms associated with severity of coronavirus disease 2019 (COVID-19): a pooled analysis. Intern Emerg Med. 2020. 10.1007/s11739-020-02329-9. 10.1007/s11739-020-02329-9 PMC7164520 32303970

[3] Shi X, Gong E, Gao D, Zhang B, Zheng J, Gao Z (2005). Severe acute respiratory syndrome associated coronavirus is detected in intestinal tissues of fatal cases. Am J Gastroenterol.

[4] Zhou J, Li C, Zhao G, Chu H, Wang D, Yan HH (2017). Human intestinal tract serves as an alternative infection route for Middle East respiratory syndrome coronavirus. Sci Adv.

[5] Darnell MER, Subbarao K, Feinstone SM, Taylor DR (2004). Inactivation of the coronavirus that induces severe acute respiratory syndrome, SARS-Co-1. J Virol Methods.

[6] Freedberg DE, Lebwohl B, Abrams JA (2014). The impact of proton pump inhibitors on the human gastrointestinal microbiome. Clin Lab Med.

